# A high-throughput assay for quantitative measurement of PCR errors

**DOI:** 10.1038/s41598-017-02727-8

**Published:** 2017-06-02

**Authors:** Dmitriy A. Shagin, Irina A. Shagina, Andrew R. Zaretsky, Ekaterina V. Barsova, Ilya V. Kelmanson, Sergey Lukyanov, Dmitriy M. Chudakov, Mikhail Shugay

**Affiliations:** 1 Shemyakin-Ovchinnikov Institute of Bioorganic Chemistry RAS, Moscow, Russia; 2 Pirogov Russian National Research Medical University, Moscow, Russia; 3 Evrogen JSC, Moscow, Russia; 4 Skolkovo Institute of Science and Technology, Moscow, Russia; 5 Central European Institute of Technology, Masaryk University, Brno, Czech Republic

## Abstract

The accuracy with which DNA polymerase can replicate a template DNA sequence is an extremely important property that can vary by an order of magnitude from one enzyme to another. The rate of nucleotide misincorporation is shaped by multiple factors, including PCR conditions and proofreading capabilities, and proper assessment of polymerase error rate is essential for a wide range of sensitive PCR-based assays. In this paper, we describe a method for studying polymerase errors with exceptional resolution, which combines unique molecular identifier tagging and high-throughput sequencing. Our protocol is less laborious than commonly-used methods, and is also scalable, robust and accurate. In a series of nine PCR assays, we have measured a range of polymerase accuracies that is in line with previous observations. However, we were also able to comprehensively describe individual errors introduced by each polymerase after either 20 PCR cycles or a linear amplification, revealing specific substitution preferences and the diversity of PCR error frequency profiles. We also demonstrate that the detected high-frequency PCR errors are highly recurrent and that the position in the template sequence and polymerase-specific substitution preferences are among the major factors influencing the observed PCR error rate.

## Introduction

Polymerase error rate is a critical factor affecting the accuracy of a wide range of molecular biology techniques, including DNA cloning^1^, PCR-based single-nucleotide polymorphism (SNP) and mutation detection^2^ and library preparation for high-throughput sequencing^3, 4^. Correct assessment of polymerase fidelity is therefore a prerequisite for obtaining robust and reproducible results in a wide variety of studies^5^.

The earliest PCR fidelity assay was a cloning-based technique^6^, which was successfully used to assess the fidelity of various DNA polymerase enzymes^7^. Techniques based on direct sequencing of PCR cloning products are commonly used at present^8^. The main drawback of these assays is that they are not very scalable: sequencing individual clones is laborious, and it is not feasible to gather a sample of errors large enough to comprehensively describe error patterns and frequency distribution. The latter information is highly valuable due to the remarkable difference in individual error frequencies, as discussed below.

High-throughput sequencing-based methods can in theory overcome the problem of cloning-based methods, but relatively poor sequencing quality turns out to be the limiting factor when quantifying polymerase accuracy. With typical quality scores of Phred 30–40, the sequencing error rate of Illumina instruments is more than an order of magnitude higher than the error rates of high-fidelity polymerases. Moreover, the sequencing quality and error rate are nucleotide-specific^9^, leading to additional biases when attempting to estimate PCR error rate from sequencing data. It was previously pointed out^10^ that the Roche 454 platform can be used to overcome these limitations due to its low substitution error rate^11^. However, this instrument’s low read yield and variable read length make it unfeasible to conduct a comprehensive study involving multiple polymerases and template molecules. Accordingly, the original study with the Roche 454 system relied on single template molecules obtained by limiting dilution. This led to an indirect per-base-per-cycle error rate estimate, drawn from a two-step PCR with 60 cycles separated by a limiting dilution step. Moreover, this setup cannot fully rule out the presence of residual sequencing errors, as the overall PCR error rate was estimated to be 0.06%^10^, while 454 sequencing can only reliably call variants with greater than 0.1% frequency^11^.

To overcome this limitation, we have turned to a technique based on unique molecular identifiers (UMI)^12–14^, which makes it possible to trace individual DNA templates throughout different library preparation stages. This technique has been successfully combined with high-throughput sequencing in various configurations for a wide range of applications that require precise quantification of rare variants^13, 15, 16^. Our template DNA molecules are subjected to two rounds of PCR amplification. By introducing a sampling bottleneck after the first PCR reaction, we were able to discriminate errors introduced during that PCR procedure from those that are introduced in subsequent amplification and sequencing steps. Using this approach, we have observed specific PCR error patterns that are recurrent and are highly specific for each polymerase. Our results reveal the complexity of the frequency distribution of individual PCR errors, which vary greatly across substitution types and positions in the template and cannot be evaluated with a single mean error rate estimate. Using a DNA library that was not subjected to PCR amplification prior to sequencing, we demonstrate that the errors associated with high sequencing quality scores resemble the PCR error pattern, providing evidence for bridge-PCR amplification errors in high-filtered high-throughput sequencing data. Our analysis also shows that the position in the template sequence and polymerase-specific substitution preferences are among the major factors influencing PCR error rate.

## Results

### A high-throughput sequencing assay for PCR error quantification

**Figure 1:**
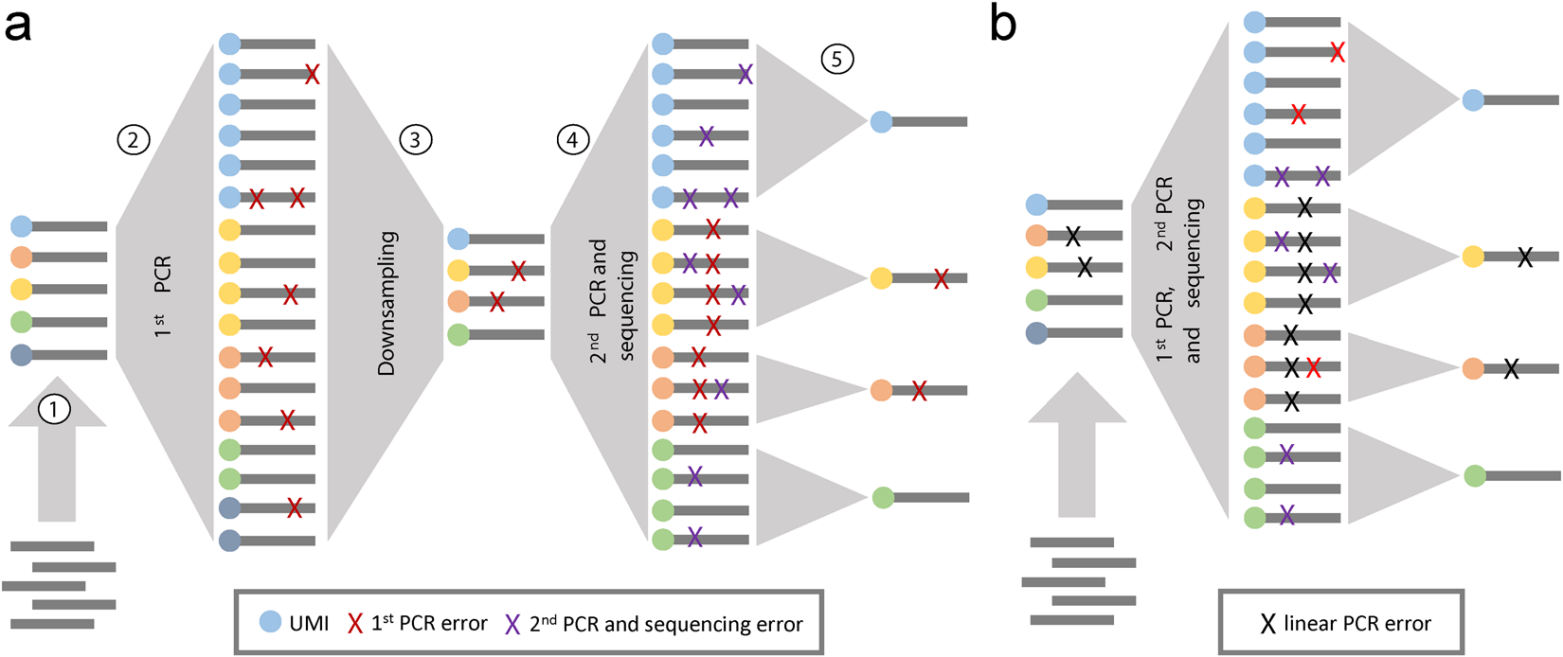
Experimental design for UMI-based evaluation of PCR errors. (**a**) Schematic representation of our five-stage experiment to evaluate errors introduced during conventional PCR amplification. DNA molecules were tagged with unique molecular identifiers (UMI) using linear amplification (1), followed by PCR amplification with various polymerases (2). We predict approximately 1.3 × 10^5^-fold amplification given a PCR efficiency of 1.8 and 20 cycles of PCR. Next, we performed a series of dilutions (~10^6^-fold downsampling) (3) to ensure that no more than one molecule with a given UMI tag is subjected to a second round of PCR (4). Finally, the PCR-amplified pool was subjected to high-throughput sequencing. The downsampling step guarantees that errors produced during the first PCR will represent the more frequent variant within reads tagged with the same UMI after sequencing, clearly distinguishing them from errors introduced at subsequent stages. Sequencing reads were processed (5) to ensure that only errors from the first PCR are retained, while those arising during the second PCR step and sequencing errors are filtered. (**b**) A simpler protocol for evaluating the error rate of the linear amplification stage used for UMI attachment. Such errors are indistinguishable from those arising during the first PCR step in the experimental setup depicted in (**a**), and the results from this simpler procedure are used to adjust the error rates inferred for conventional PCR amplification.

Our protocol involves five steps (Fig. 1a). We began by tagging each input template molecule (step 1) with a random 14-mer nucleotide tag (UMI) in a linear amplification procedure, and then performing PCR amplification (step 2) with one of nine different assayed polymerases (see Materials and Methods and Table 1). This first PCR step consisted of 20 (25 for Phusion polymerase) cycles starting from a single-strand template; assuming the PCR efficiency to be 1.8 (ref. 17), we would expect ~10^5^–10^6^-fold amplification of the input DNA.

**Table 1:** Error rate estimates from two independent experiments. The table shows the number of mismatches that remain after consensus sequence assembly for reads tagged with the same UMI, the total number of unique UMI tags observed, and error rate estimates for 20 (25 for Phusion) cycles of PCR amplification with a 150-bp template. Error rate estimates are provided as the number of erroneous bases per template base per cycle, with 95% confidence intervals (CI) calculated using normal approximation for binomial proportions. Error rate^LA^ represents estimates for the linear amplification step, while Error rate^corr^ represents the estimate of conventional PCR error rate after correcting for linear amplification errors.

Polymerase*	**Exp**.	**Error count**	**UMI tags**	**Error rate, x10** ^**−5**^ **[95% CI]**	**Error rate** ^**LA**^ **, x10** ^**−5**^	**Error rate** ^**corr**^ **, x10** ^**−5**^ **[95% CI]**
Encyclo	1	24557	185560	4.30 [4.25, 4.35]	12.79	3.68 [3.63, 3.73]
Encyclo	2	14211	101516	4.55 [4.48, 4.62]		3.93 [3.86, 3.99]
Kapa HF	1	339	7876	1.40 [1.25, 1.55]	8.17	1.00 [0.88, 1.13]
Kapa HF	2	2519	57052	1.44 [1.38, 1.49]		1.04 [0.99, 1.08]
Phusion	1	30	1348	0.72 [0.47, 0.98]	4.99	0.48 [0.27, 0.69]
Phusion	2	23	1351	0.55 [0.33, 0.78]		0.31 [0.14, 0.48]
SD-HS	1	6714	33076	6.60 [6.46, 6.74]	26.89	5.29 [5.16, 5.42]
SD-HS	2	10362	58518	5.76 [5.66, 5.86]		4.45 [4.36, 4.54]
SNP-detect	1	457	13870	1.07 [0.97, 1.17]	5.04	0.83 [0.74, 0.91]
SNP-detect	2	848	32310	0.85 [0.80, 0.91]		0.61 [0.56, 0.66]
Taq-HS	1	1875	15137	4.03 [3.86, 4.20]	18.36	3.13 [2.98, 3.29]
Taq-HS	2	3113	24082	4.20 [4.07, 4.34]		3.31 [3.18, 3.43]
Tersus-buffer1	1	2550	46927	1.77 [1.70, 1.83]	5.98	1.48 [1.41, 1.54]
Tersus-buffer1	2	5504	154226	1.16 [1.13, 1.19]		0.87 [0.84, 0.89]
Tersus-buffer2	1	6282	130891	1.56 [1.52, 1.60]	5.1	1.31 [1.28, 1.35]
Tersus-buffer2	2	1299	30683	1.38 [1.30, 1.45]		1.13 [1.06, 1.19]
TruSeq	1	312	14164	0.72 [0.64, 0.79]	4.1	0.52 [0.45, 0.58]
TruSeq	2	362	16705	0.70 [0.63, 0.78]		0.50 [0.44, 0.57]
KTN	1	16802	132733	4.12 [4.06, 4.17]	12.92	3.49 [3.43, 3.54]
KTN	2	6298	44331	4.62 [4.51, 4.73]		3.99 [3.89, 4.09]

^*^Encyclo (Evrogen JSC), Kapa HiFi PCR Kit (Kapa Biosystems), Phusion High-Fidelity DNA Polymerase (NEB), SD-HS^29^, SNP-detect, HS Taq, Tersus in buffer 1 (Mg^2+^ 3.5 mM, Ph 8.5), Tersus in buffer 2 (Mg^2+^ 2.5 mM, Ph 8.0) (Evrogen JSC), TruSeq Custom Enrichment Kit (Illumina), KTN (KlenTaq N polymerase)^30^.

Next, we performed a series of dilutions to remove PCR duplicates generated during the 1st PCR step (step 3), ensuring that at most a single DNA molecule is sampled for each input template. These are then subjected to a second PCR step (step 4) of 22–29 cycles, followed by high-throughput sequencing analysis (step 5). Because of the dilution procedure, all sequencing reads with the same UMI tag are derived from copies generated during the second PCR step, and the most frequently detected sequence variant within the sequencing read group will represent the exact sequence that was sampled from the first PCR reaction. This strategy allows us to correct errors associated with the second PCR and sequencing steps by assembling a majority consensus sequence while preserving errors produced at previous stages^13, 15^. The sequencing error correction in this case is trivial: for a sample of UMI tags each covered by five 100-bp-long reads and a sequencing quality of Phred 30 (0.1% errors per read at a given position), the probability of observing a sequencing error that is present in at least 3 out of 5 reads at the same position is less than 1 per million UMI tags. We then estimated the resulting error rate for each polymerase as the ratio of the number of errors in the consensus sequences to the product of the total number of UMI tags (templates), the template length, and the number of cycles in the first PCR step.

We additionally ran the same protocol without the dilution step between the first and second PCR (Fig. 1b), which allowed us to correct all PCR and sequencing errors except those introduced at the linear amplification stage. Notably, we found that the frequency of these linear amplification-associated errors is 5 ± 1 times higher (Table 1) than the per-cycle error rate of the subsequent PCR amplification. The latter can be attributed to two factors: higher dNTP concentration that increases the error rate^7^, and differences in polymerase efficiency, as the per-cycle error rate is inversely proportional to efficiency^18^. The dNTP concentration is greatly depleted over the course of 20 cycles^19^, and thus the mean dNTP concentration during the first PCR reaction step is smaller than at the start of the reaction. We observed the highest (~8-fold) and lowest (~2.9-fold) error rate ratio between the linear amplification and PCR reaction steps for the Phusion and Encyclo polymerase samples, respectively, which had the lowest and highest UMI tag count (Table 1) produced from the same starting amount of DNA, making these polymerases the least and most efficient, respectively. The expected number of linear amplification errors was then subtracted from the total number of errors for each sample to produce a final error rate estimate.

Our error rate estimates (Table 1) were in good agreement with previously published data^8^, and highly consistent between two independent experiments (Table 1, Supplementary Figure 1, R = 0.97, P = 3 × 10^−6^). We observed a clear peak in the number of reads tagged with the same UMI for each polymerase (Supplementary Figure 2), suggesting that almost all of the individual molecules that were sampled after the first PCR step are found in the resulting sequencing read dataset. The correlation between individual UMI coverage and the number of cycles in the second PCR step (Supplementary Figure 3, R = 0.91, P = 2 × 10^−8^) further confirms that we were successful at implementing the sampling bottleneck, which ensures that only errors generated by the second PCR step and sequencing will be corrected by consensus assembly, leaving the errors from the first PCR step intact. The number of observed UMIs tags (Table 1) was highly variable across polymerases due to differences in efficiency. The lowest value was observed for Phusion polymerase (2,699 in two experiments combined), whereas Encyclo polymerase produced the highest value (287,076 in two experiments combined). On average, there were 110,236 UMI tags per PCR assay in two experiments combined.

It is necessary to note that the Phusion polymerase yielded very few starting molecules despite having the highest number of amplification cycles and largest amount of input DNA (see Supplementary Table 3). This can be attributed to low polymerase efficiency, and can be dealt with by substantially increasing the amount of input DNA. Thus, careful protocol adjustments should be performed when dealing with low-efficiency polymerases. It was not feasible to study individual errors and nucleotide patterns for the amount of input molecules observed with Phusion, and this polymerase was therefore excluded from further analysis.

### Substitution type preferences and unique fingerprint of PCR errors

We next analyzed the features of PCR errors inferred from datasets obtained as described above in the context of substituted nucleotide types, and compared them across tested polymerases. The strong preference for transitions (purine-purine and pyrimidine-pyrimidine substitutions) over transversions (purine-pyrimidine substitutions) in DNA polymerase errors has been extensively described, and was previously demonstrated for both DNA replication in living cells^20^ and PCR reaction products^8^, although some notable counter-examples do exist^21^.

**Figure 2:**
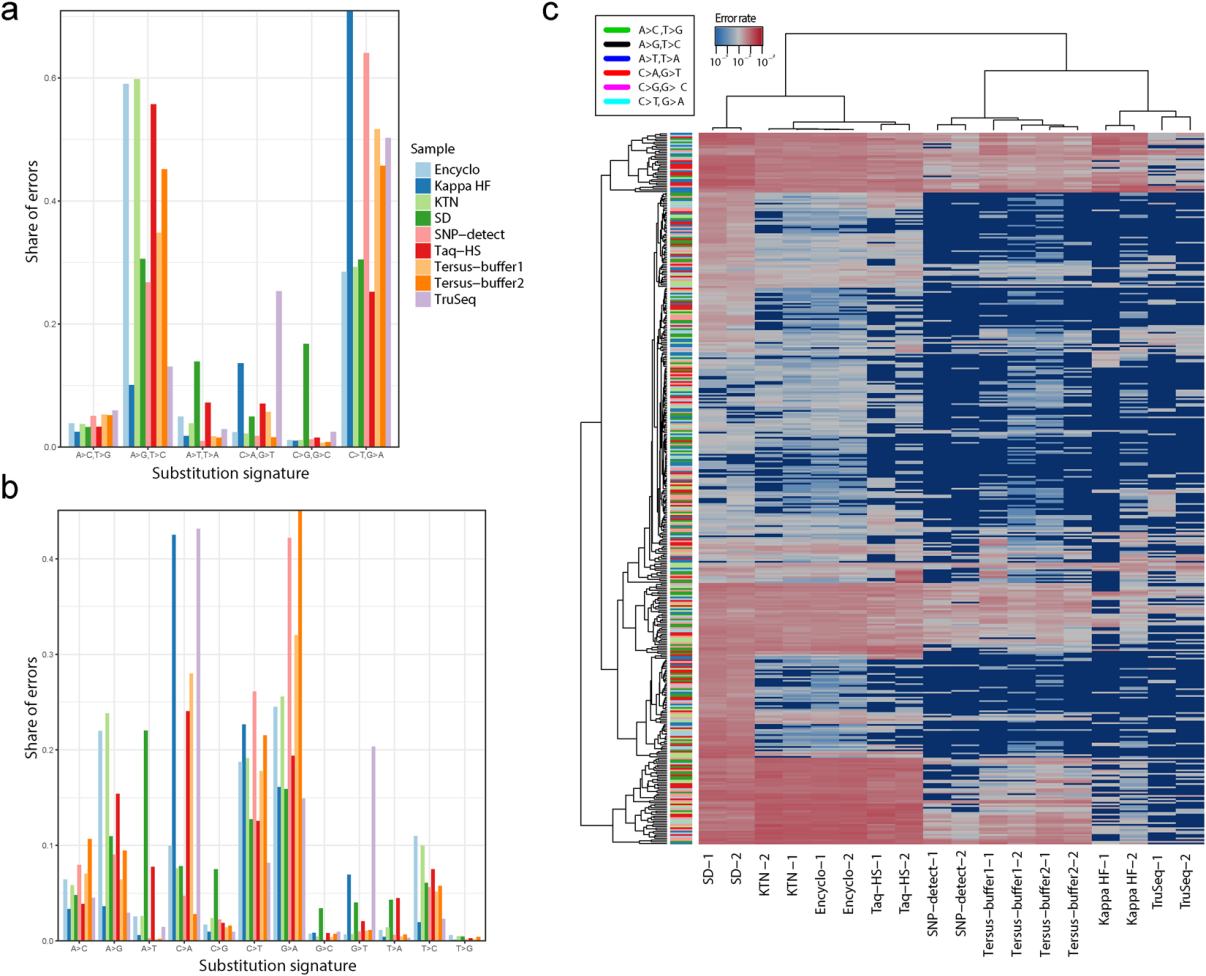
Substitution type preferences and unique error profiles of different polymerases. (**a**) Share of each substitution type produced during 20 PCR cycles (top) and one cycle of linear amplification (bottom) in each sample. Substitution frequencies were normalized to the ratio of the corresponding base type in the template sequence. Note that linear amplification preserves the strand information and thus allows us to resolve all twelve possible substitution types. (**b**) Hierarchical clustering of PCR error profiles, computed as the frequency of each of three possible substitutions at each template position produced in two independent experiments. Correlation and Euclidean distance were used to cluster polymerases (columns) and substitutions (rows), respectively. The color panel at left shows substitution type, as represented in the legend at top.

We computed ratios for A > C/T > G, A > G/T > C, A > T/T > A, C > A/G > T, C > G/G > C and C > T/G > A substitutions by determining the share of corresponding substitutions in each sample (Fig. 2a, top). The analysis of PCR errors produced during 20 PCR cycles shows that all polymerases display a strong transition error preference (Table 2), but fall into two categories based on the dominant substitution type: C > T and G > A for Kapa HF, SNP-detect, Tersus-buf1, Tersus-buf2 and TruSeq, and A > G and T > C for Encyclo, SD-HS, Taq-HS and KTN. We also analyzed error spectra from linear amplification, which has the advantage of preserving the strand information and therefore allows us to distinguish all twelve substitution types (Fig. 2a, bottom). Interestingly, at this level, several polymerases showed a dominant transversion error type: A > T for SD-HS, and C > A for Kapa HF, Taq-HS, and TruSeq (Table 3). Another peculiar observation is that 20% (the second most common error type) of TruSeq errors were G > T transitions, which are extremely rare for other polymerases. SD-HS, being the most error-prone polymerase, also showed the most uniform error spectrum.

**Table 2:** Frequency of substitution types produced during 20 PCR cycles.

Polymerase	A > C, T > G	A > G, T > C	A > T, T > A	C > A, G > T	C > G, G > C	C > T, G > A
Encyclo	4%	**59%**	5%	2%	1%	29%
Kapa HF	2%	10%	2%	14%	1%	**71%**
SD-HS	3%	**31%**	14%	5%	17%	30%
SNP-detect	5%	27%	1%	2%	1%	**64%**
Taq-HS	3%	**56%**	7%	7%	2%	25%
Tersus-buffer1	5%	35%	2%	6%	1%	**52%**
Tersus-buffer2	5%	45%	2%	2%	1%	**46%**
TruSeq	6%	13%	3%	25%	3%	**50%**
KTN	4%	**60%**	4%	2%	1%	29%

Relative frequency of each of six substitution types that can be inferred without strand information across all errors produced by each polymerase. We used pooled data from two independent experiments to construct the table, with frequencies normalized to the ratio of the corresponding base in the template sequence. The most frequent substitution for each polymerase is shown in bold.

**Table 3:** Frequency of substitution types produced during linear amplification.

Polymerase	A > C	A > G	A > T	C > A	C > G	C > T	G > A	G > C	G > T	T > A	T > C	T > G
Encyclo	6%	22%	3%	10%	2%	19%	**25%**	1%	1%	1%	11%	1%
Kapa HF	3%	4%	1%	**42%**	1%	23%	16%	1%	7%	0%	2%	0%
SD-HS	5%	11%	**22%**	8%	7%	13%	16%	3%	4%	4%	6%	0%
SNP-detect	8%	9%	0%	5%	2%	26%	**42%**	0%	1%	1%	6%	0%
Taq-HS	4%	15%	8%	**24%**	2%	13%	19%	1%	2%	4%	8%	0%
Tersus-buffer1	9%	6%	0%	22%	1%	18%	**38%**	1%	1%	1%	4%	0%
Tersus-buffer2	10%	10%	0%	6%	2%	22%	**40%**	1%	1%	1%	7%	0%
TruSeq	5%	3%	1%	**43%**	1%	8%	15%	1%	20%	0%	2%	0%
KTN	6%	24%	3%	8%	2%	19%	**26%**	0%	1%	1%	10%	0%

Relative frequency of each of twelve possible substitution errors produced by each polymerase. We used pooled data from two independent experiments to construct the table, with frequencies normalized to the ratio of the corresponding base in the template sequence. The most frequent substitution for each polymerase is shown in bold.

While there are some general similarities between error spectra across polymerases, we decided to test whether each of them has a unique error fingerprint. We computed error profiles as the frequency of each of three possible substitutions at each template position and applied hierarchical clustering to these profiles (Fig. 2c). Interestingly, the clustering produced matching error profiles for each of the polymerases in two independent experiments. We identified four discrete clusters, with co-clustering of Kapa HF/TruSeq, SNP-detect/Tersus-buf1/Tersus-buf2 and KTN/Encyclo/Taq-HS, with SD-HS as an outlier. These clusters are in partial agreement with the substitution type preferences shown in Table 3: Kapa HF/TruSeq are both C > A prone, SNP-detect/Tersus-buf1/Tersus-buf2 show high G > A rate, and SD-HS is distinctive in terms of its dominant A > T substitution type. On the other hand, there was no evidence for clustering of error profile units (frequency value for a given position and substitution type) by substitution type, suggesting that the unique fingerprint of each polymerase is produced in a context-specific manner and is not completely defined by differences in the share of errors having certain substitution types.

### Complexity of frequency distribution and recurrence of individual PCR errors

**Figure 3:**
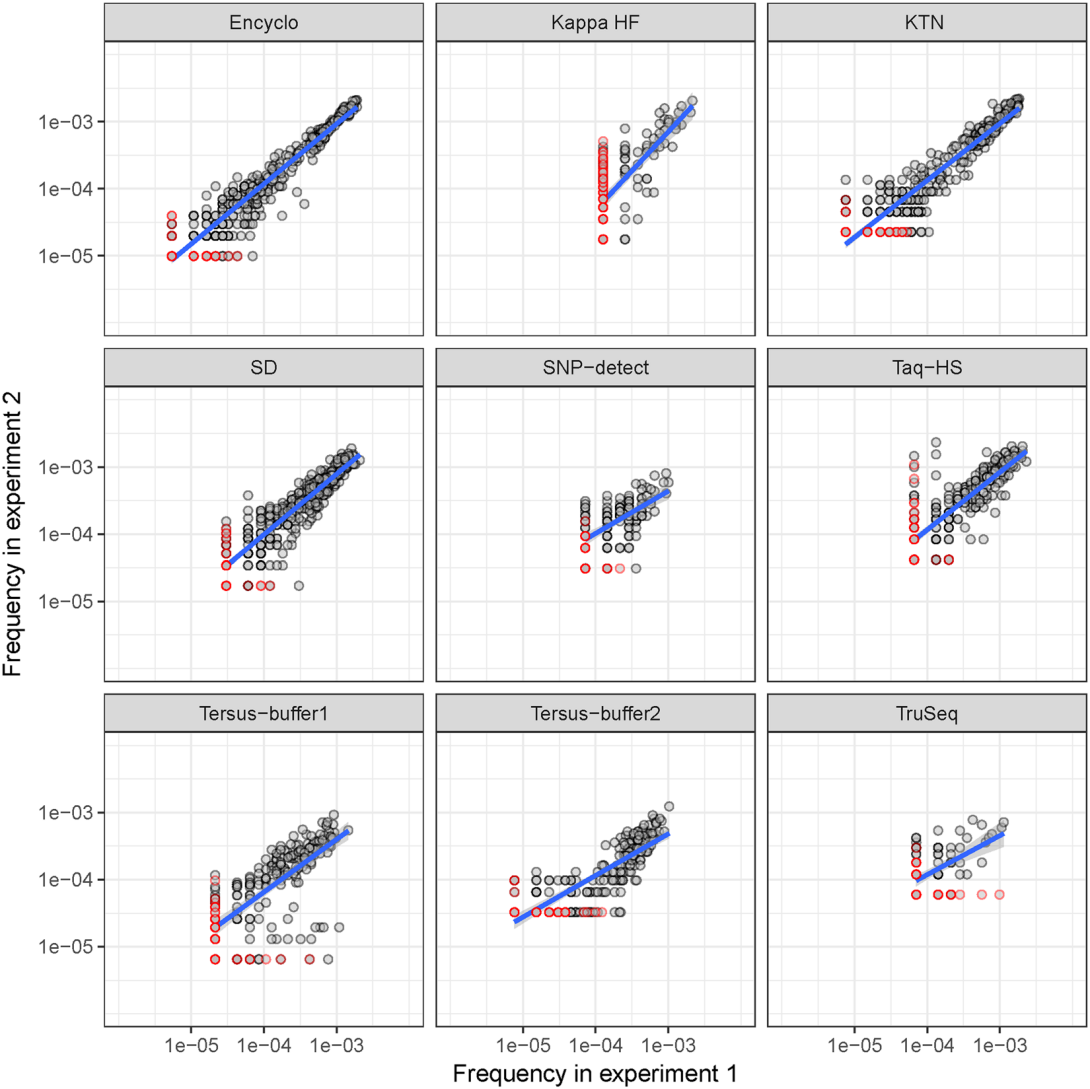
Recurrent high-frequency errors in two separate experiments. Scatter plots of individual substitution frequencies obtained from two independent experiments with 20 cycles of PCR. Red circles indicate PCR errors that were not detected in one of the experiments; in this case, their frequency is assumed to be 1 divided by the corresponding region coverage. Linear fitting is shown by blue lines.

Practical applications require careful assessment of background noise introduced by PCR—namely, the recurrence of PCR errors and their individual frequencies. As seen in Fig. 3, we detected high-frequency errors at a similar rate for all polymerases in 20 cycle PCR reactions in two independent experiments. These recurrent errors can reach a frequency of >0.1%, putting them in the sensitivity range of state-of-art assays for circulating tumor DNA detection^22^. Replicate experiments^23^ are unfeasible in this scenario, and therefore appropriate PCR error models and other techniques such as the UMI approach should be used instead for ultra-deep sequencing.

**Figure 4:**
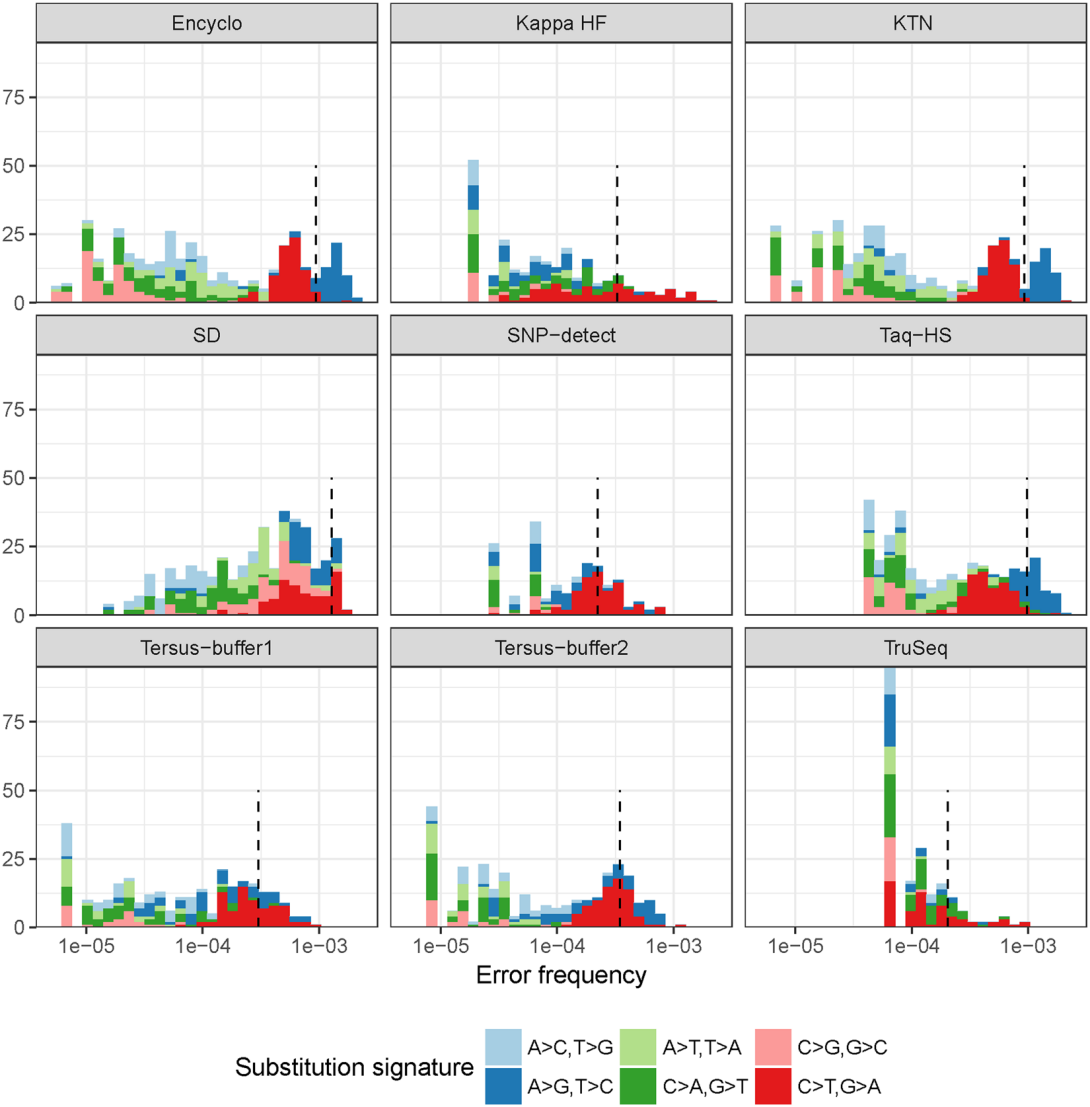
Frequency distribution of individual PCR errors. The mixture of individual error (grouped by position and substitution type) frequency histograms for each substitution type (shown by color) in each sample. Dashed line shows the mean PCR error rate estimate after 20 PCR cycles.

It has been previously demonstrated that mutations induced in living cells by DNA polymerase are distributed in a highly non-uniform manner, and genomes contain hot-spot regions with high mutation frequency^24^. This frequency variance is an important factor to take into account when building statistical models of PCR errors. The resolution provided by our protocol allowed us to study the distribution of individual error frequencies and substitution types, and their differences across polymerases. As can be seen from Fig. 4, the histogram of error frequencies is a complex mixture of distributions corresponding to different substitution types and cannot be described with a single mean error rate value for each polymerase. In some cases, such as C > T, G > A and A > G,T > C substitutions in KTN and Encyclo samples, these distributions are evident in the mixture.

Figure 4 highlights the important fact that describing error frequencies with a generic polymerase fidelity estimate can be highly misleading. First, there is a strong variance in the frequency distribution across different substitution types. Moreover, in many applications, such as detection of rare mutations in tumor and viral genomes^13, 25^ and the characterization of T- and B-cell receptor sub-variants^15^, the accuracy of variant calling is limited by the probability of an error with a given substitution type occurring at a given position. If this probability is not known precisely, the distribution of error frequencies for the corresponding substitution type and the worst case of high-frequency errors should be taken into account instead of relying on the average PCR error rate.

### Evidence of residual PCR errors in quality-filtered sequencing data from an unamplified library

Illumina sequencing involves a bridge PCR step, where each solid phase-immobilized molecule is amplified to ~1,000 copies^9^. A PCR error at the initial step of the cluster generation process (or at the second step, in case of inefficient amplification) can produce a dominant erroneous variant that will be read from the cluster^13^. These errors can limit the accuracy of ultra-deep sequencing, as they are not eliminated by increasing the sequencing quality or discarding low-quality base calls. To study the errors introduced at this step, we sequenced a cloned DNA library that was not subjected to PCR amplification prior to sequencing, and which contained the same template that was used for PCR error quantification. Sequencing errors were then filtered by raising the sequencing quality threshold. We expected that with an increase in quality threshold, the bridge-PCR error signature would become dominant as sequencing errors diminish.

**Figure 5:**
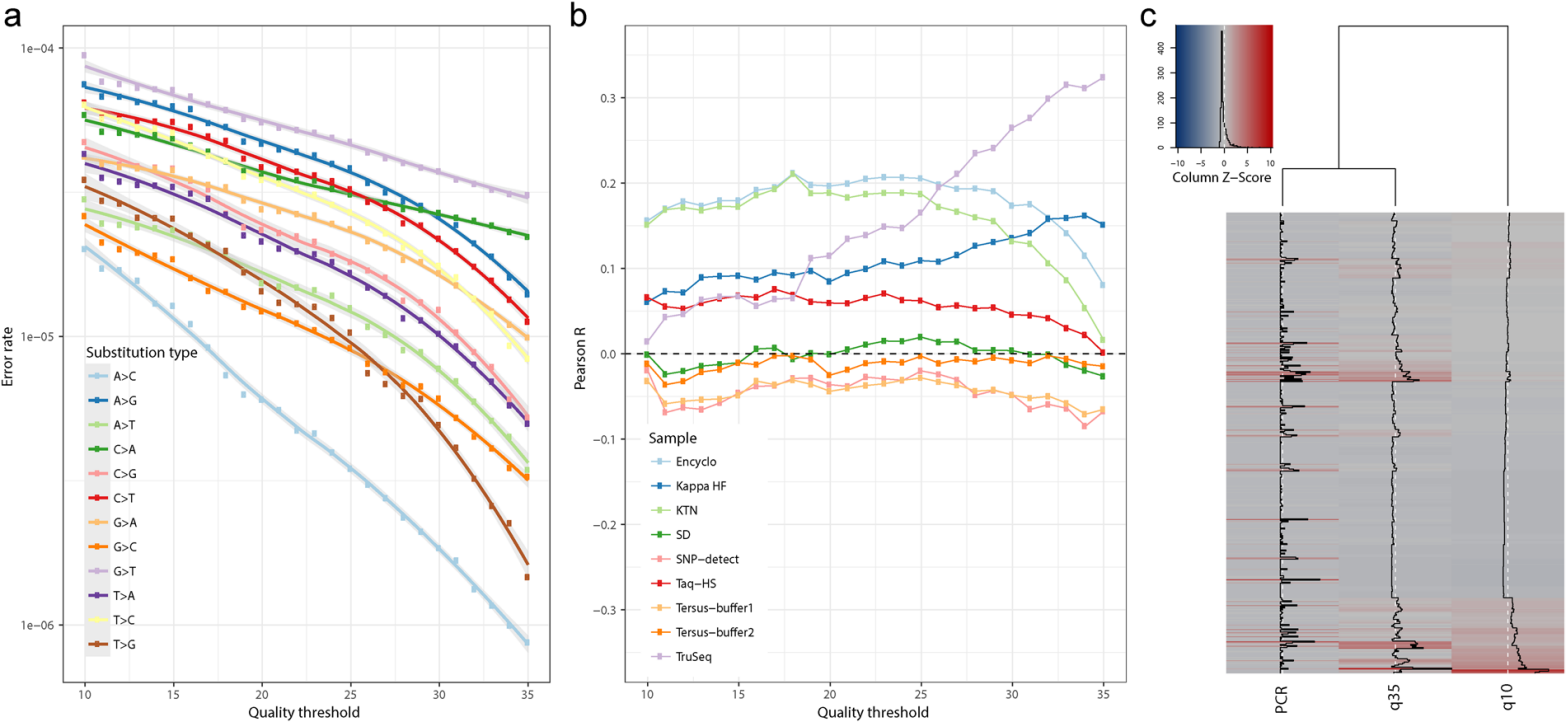
Evidence for bridge PCR errors in quality-thresholded sequencing data. (**a**) Erroneous variants from an unamplified control were thresholded by sequencing quality, and the error rate of each substitution type was computed as the ratio of corresponding errors to the number of reads that have quality greater than or equal to the threshold. The overall error rate was between 10^−3^–10^−4^ when no filtering was applied, in keeping with the fact that most bases in this experiment have a sequencing quality score of Phred 35. Errors characteristic for the TrueSeq polymerase signature after one cycle of linear amplification (C > A, G > T) become dominant beyond the Phred 30 quality threshold. (**b**) Correlation between the PCR error profile (*i.e*., frequencies of substitutions at each template position) and errors in the amplification-free control at various sequencing quality thresholds. (**c**) Hierarchical clustering of error profiles produced by TrueSeq and profiles obtained at Q10 and Q35 quality thresholds with an unamplified control.

Indeed, as can be seen in Fig. 5a, C > A and G > T errors become dominant beyond a quality threshold of Q30, a signature that closely resembles the one observed in the error spectrum of the TruSeq and Kapa HF polymerases. For further validation, we computed the correlation between the error profiles of each linear amplification assay and the quality-filtered sequencing data. Figure 5b shows that the correlation between the sequencing error profile and the TruSeq error profile steadily increases with the rising quality threshold, whereas no such correlation is observed for the error profiles from other polymerases (except for a minor trend observed for Kapa HF). Moreover, clustering of the TruSeq error profile and sequencing error profiles at quality thresholds of Q10 and Q35 shows that Q35 errors appear more similar to those produced by TruSeq than those observed at Q10 (Fig. 5c). Overall, this provides evidence for persistent bridge-PCR sequencing errors that can limit the precision of sequencing, especially for high-quality Illumina HiSeq datasets.

### Summarizing the contributions of different factors that affect PCR error rate

So far, we have described individual polymerase substitution preferences, leaving aside the context and positioning of PCR errors. We next set out to build a model of PCR error rate that incorporates all of these aforementioned factors. We have used linear PCR data as it contains data from a single strand in contrast to 20 cycle PCR reaction and allows to distinguish all four bases and the exact position on the template with respect to PCR primer.

**Figure 6:**
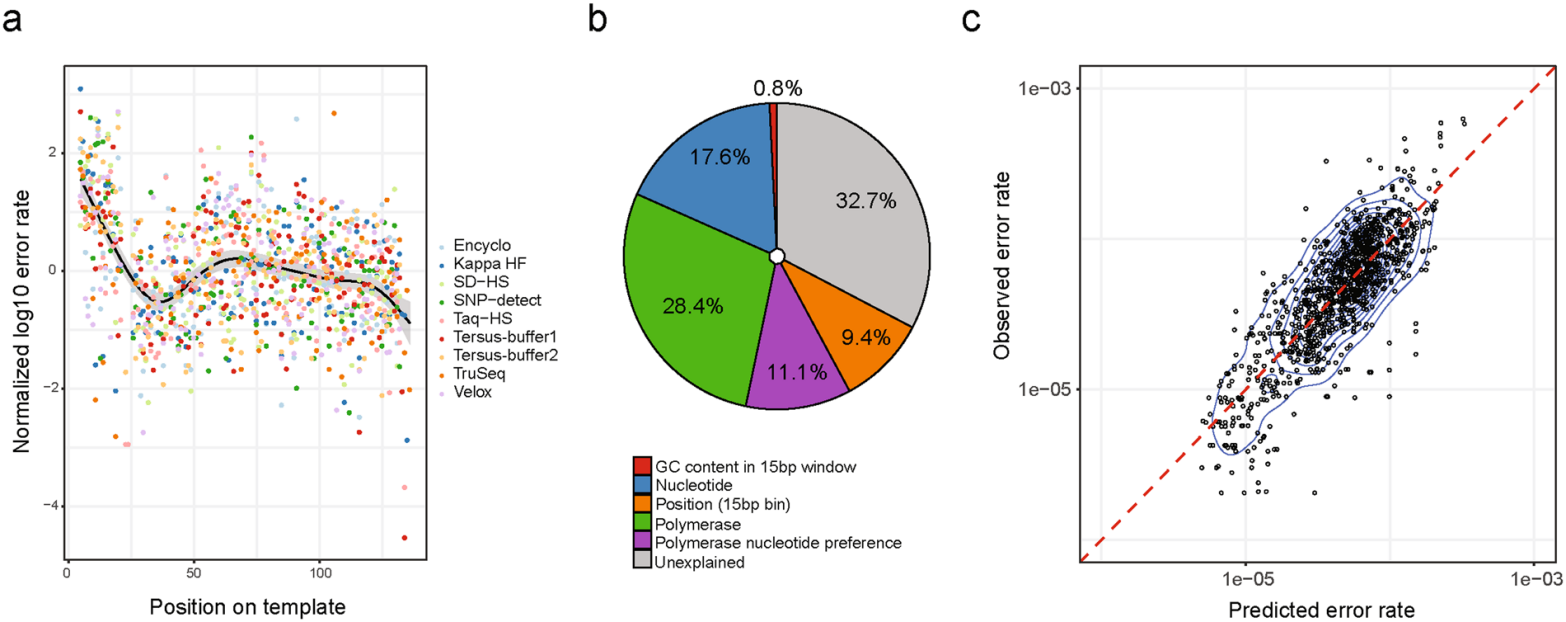
Template position and other factors affecting PCR error rate. (**a**) Normalized log10 error rate for each template position. The normalization was performed by scaling log-transformed error rate values for each nucleotide type and polymerase to zero mean and unit standard deviation. The Spearman correlation coefficient between normalized error rate and position is R = −0.21 (P < 10^−11^). (**b**) Percent of variance in error rate explained by GC content, nucleotide type, polymerase, polymerase-specific nucleotide preference, and position. GC content was computed within a 15-bp window centered on the erroneous base. The position variable was computed by splitting the template sequence into non-overlapping 15-bp bins, where the index of the bin corresponding to each erroneous base was used as a variable. Explainable variance was computed using ANOVA of a linear model that includes all of the aforementioned factors. (**c**) Observed error rate plotted against the error rate predicted using the linear model. The correlation between the observed and predicted error rate was R = 0.63 (P < 10^−116^).

In order to examine the error rate across different parts of the template sequence, we normalized the error rate as follows: log-transformed error rate values for each sample and template base type were scaled to have zero mean and unit standard deviation. We observed a complex trend of error rate change with respect to position in the template, suggesting that some portions of the template are more error-prone, even when controlling for polymerase type and substituted nucleotide type (Fig. 6a).

We next fitted a linear model that explains the log-transformed error rate using error position on the template, GC content of the region surrounding the error, substituted nucleotide type, polymerase type and polymerase-specific substitution preference (*i.e*., interaction between substituted nucleotide- and polymerase-related factors). To account for the position factor, we divided the template into 15-bp non-overlapping bins and used the bin index as a categorical variable in our model. The contribution of each factor to the observed PCR error rate was then assessed using ANOVA (Fig. 6b). The type of polymerase explained 28.4% (P < 10^−132^) of variance, followed by substituted nucleotide type (17.6%, P < 10^−94^), polymerase-specific substitution preference (11.1%, P < 10^−49^), position (9.4%, P < 10^−47^) and GC content (0.8%, P < 10^−6^). Interestingly, polymerase-related factors explained most of the known variance in PCR error rate (39.5%), and this relatively simple model explained as much as 67% of the overall error rate variance. On the other hand, the surrounding GC content has little influence on error rate, suggesting a non-trivial relationship between error rate and nucleotide context. The comparison of observed and fitted PCR error rates is shown in Fig. 6c; fitted values display good correlation with the observed error rate (R = 0.63, P < 10^−116^).

## Discussion

Given the widespread use of high-throughput sequencing assays that include PCR amplification steps for high-precision tasks such as detection of ultra-rare mutations, it is critical to develop proper methodology to quantify errors and artefacts that arise in the process of sequencing library preparation. Analysis of high-throughput sequencing data mostly relies on quality scores to measure the accuracy of variant calling, but it has become evident that even when sequencing errors are efficiently eliminated the data is not error-free, and in fact contains recurrent high-frequency PCR errors that undermine accuracy^15, 26^. This is further supported by our findings from this work (Figs 3–5), and comprehensive characterization of PCR error rate profiles will be a prerequisite for further development of methods such as rare mutation detection in tumor and viral genomes or monitoring of circulating tumor DNA.

The novel high-throughput, UMI-based PCR error rate assay described in the present work efficiently overcomes the limitations of previous techniques, generating substantial PCR error statistics from a large population of individual DNA template molecules and several polymerases using a single HiSeq lane. With this method, we were able to reveal the complexity of polymerase error profiles and highlight non-uniform error rate distributions that are apparently fundamental characteristics of individual polymerase enzymes. While high-fidelity polymerases have much lower error rates on average than their error-prone counterparts, we still observed some overlap between them at the level of individual error frequencies (Fig. 4). These high-frequency errors, being recurrent (Fig. 3) and having a rate of more than 10^−4^ (corresponding to an extremely high Phred quality score of 40) could be easily mistaken for real variants. However, the pattern of those high-frequency errors is in good agreement with the substitution preferences of the corresponding polymerase enzyme (Fig. 2a and Tables 2 and 3); if properly quantified, these error profiles can be used to correct confidence scores for variant calls.

The results obtained in the present study can be used to develop statistical models of PCR errors that will improve the accuracy of existing variant-calling software. Such models will be extremely useful for certain high-precision applications, such as the detection of rare somatic mutations^26, 27^. One of the limitations of the current work is that it relies on a generic PCR efficiency value to estimate error rates. However, with proper calibration, the current protocol can be employed to quantify polymerase efficiency.

The protocol described here is relatively simple to implement and the resulting data can easily be interpreted without sophisticated bioinformatic analysis. By taking advantage of the scalability of the current protocol and starting from a more complex library that incorporates multiple distinct regions, one can quantify amplification biases, infer the context shaping the unique fingerprint of the polymerase (Fig. 2b), explain differences in the PCR error rate across the template (Fig. 6), and ultimately reveal the landscape of PCR error hot-spots that limit the precision of current high-sensitivity methods^15, 26, 28^.

## Materials and Methods

### Preparation of UMI-labeled libraries

The 150-nt template DNA fragment, flanked by Illumina TruSeq adapters, was cloned into the pAl-TA plasmid (Evrogen, Russia). This template, cut from the plasmid, represents a ready-for-sequencing product, and was further used as an unamplified control. To control for possible cross-sample contamination in the sequencing output, nine indexed sub-variants of the control template were generated individually for each polymerase being compared (Supplementary Table 1). These were cloned into the pAl-TA plasmid and verified by Sanger sequencing. Each plasmid DNA template was further amplified in one of 10 (for each individual polymerase being tested, with the exception of Tersus polymerase which was tested two times in two different buffers) three-stage reactions (see Supplementary Table 2 for oligonucleotides used and Supplementary Table 3 for polymerase-specific reaction conditions).

#### Linear amplification

UMIs were introduced via three cycles of linear amplification with the TruSeq_NNNtest_pol oligonucleotide. Plasmid DNA template was pre-heated for 2 min at 70 °С. Linear amplification was performed in 50 μl reaction volume using one of the nine DNA polymerases being compared in the buffer provided by manufacturer. We used the following linear amplification program: 5 min at 95 °С; 3x [15 s at 95 °С, 20 s at 58 °С, 30 s at 72 °С]; 2 min at 72 °С. The product was purified using the MinElute PCR Purification Kit (Qiagen) and eluted in 11 μl of sterile water.

#### First PCR

10 μl of each linear amplification reaction product was used as a template for the PCR reaction, which was performed in a 50 μl volume using oligonucleotides TruSeqPCR_Uni-short-21 and TruSeqRev_testpol_Bridge, with the same DNA polymerase employed in the previous linear amplification step in the buffer provided by manufacturer. We used the following program: 5 min at 95 °С; 20x (25x for Phusion) [15 s at 95 °С, 20 s at 60 °С, 30 s at 72 °С]; 2 min at 72 °С.

#### Second PCR (for tracking first PCR and linear amplification errors)

2 μl of reaction product from the first PCR step were diluted with 78 μl of sterile water. 2 μl of diluted product were again diluted in 998 μl of sterile water. 2 μl of diluted product were used as a template for a second PCR reaction, performed in a 50 μl volume. TruSeq_Universal_long and TruSeq_Rev_long_Index oligonucleotides were used, introducing sample-specific indexed Illumina TruSeq adapters. Tersus DNA polymerase (Evrogen, Russia) was used for all samples. We used the following program: 5 min at 95 °С; 22–29x [15 s at 95 °С, 20 s at 60 °С, 30 s at 72 °С]; 2 min at 72 °С.

#### Second PCR (for tracking linear amplification errors only)

2 μl of reaction product from the first PCR step were diluted with 78 μl of sterile water. 2 μl of this diluted product were used as a template for a second PCR reaction, performed in a 50 μl volume. TruSeq_Universal_long and TruSeq_Rev_long_Index oligonucleotides were used, introducing sample-specific indexed Illumina TruSeq adapters. Tersus DNA polymerase (Evrogen, Russia) was used for all samples. We used the following program: 5 min at 95 °С; 14–18x [15 s at 95 °С, 20 s at 60 °С, 30 s at 72 °С]; 2 min at 72 °С.

Concentrations of the resulting PCR products were measured using a Qubit 2.0 Fluorometer (Invitrogen, USA). Products of the 10 PCR reactions, along with EcoRI-cut control template, were pooled in equimolar proportions, purified with the QIAquick PCR Purification kit (Qiagen), and stored at −20 °C before sequencing. Sequencing was performed on a single lane of an Illumina HiSeq 2500 using the 100 + 100 nt paired end kit for linear amplification-only experiments (step 3b above) and 150 + 150 nt paired end kit for linear amplification plus 20 cycle PCR experiments (step 3a above).

### Analysis of high-throughput sequencing datasets

Four datasets were generated using the protocol described above: two independent experiments measuring the linear amplification error rate and two independent experiments measuring both the linear amplification and PCR error rates from 20 cycles. Additionally, sequencing data were obtained for an unamplified library. Datasets were analyzed using the MAGERI (https://github.com/mikessh/mageri) pipeline^31^. Briefly, UMI tags were extracted and tags that were read less than five times were filtered, as these would not provide enough consensus sequence coverage to correct PCR and sequencing errors. While the majority of UMI tags filtered due to low coverage represent errors in UMI sequence, an additional round of filtering was performed by looking for UMI tags that have a similar “parent” sequence that differs by 1 or 2 mismatches and with a coverage ratio of less than 1:20 and 1:200, respectively. Consensus sequences were then assembled for reads grouped by UMI tag and were aligned to a synthetic reference. The output of the variant-calling module of MAGERI was used for further analysis. Datasets and all results reported in the text can be reproduced by running an R markdown template available at https://github.com/mikessh/polyfid (this also includes a script to process the data for the unamplified library). Note that no additional filtering was performed for called variants as, according to our estimates, all second PCR step and sequencing errors are filtered at the consensus assembly stage (see Results section).

## Electronic supplementary material

Supplementary Figures and Tables
