# Supplementary material for: A high-throughput assay for quantitative measurement of PCR errors
Source: Sci Rep. 2017 Jun 2;7:2718. doi: 10.1038/s41598-017-02727-8 (PMC5457411; doi:10.1038/s41598-017-02727-8)
Supplement: Supplementary file 1 — Supplementary Figures and Tables [file 41598_2017_2727_MOESM1_ESM.pdf]

SUPPLEMENTARY

A high-throughput assay for quantitative measurement of PCR errors

Dmitriy A. Shagin[1,2,3], Irina A. Shagina[2,3], Andrew R. Zaretsky[2,3], Ekaterina V. Barsova[1,3], Ilya V. Kelmanson[1,3], Sergey Lukyanov[1,2], Dmitriy M. Chudakov[1,4,2,5]*, Mikhail Shugay[1,2,5*]

* To whom correspondence should be addressed: chudakovdm@mail.ru, mikhail.shugay@gmail.com

[1]Shemyakin-Ovchinnikov Institute of Bioorganic Chemistry RAS, Moscow, Russia

[2]Pirogov Russian National Research Medical University, Moscow, Russia

[3]Evrogen JSC, Moscow, Russia

[4]Skolkovo Institute of Science and Technology, Moscow, Russia.

[5]Central European Institute of Technology, Masaryk University, Brno, Czech Republic.

Table S1. Control template and its sub-variants. Control template as cloned in pAl-TA plasmid is shown, Illumina adapters shown bold. Within plasmid, this template was flanked by EcoRI restriction sites. After cutting from plasmid with EcoRI, this control was used directly for Illumina sequencing, amplification free. Subvariant templates generated individually for each polymerase of comparison are shown as cloned in pAl-TA plasmid. Internal sample-specific indexes underlined. Both control template and its sub-variants cloned in pAl-TA plasmid were verified by Sanger sequencing. Sample index bases were removed during analysis.

Control template	**AATGATACGGCGACCACCGAGATCTACACTCTTTCCCTACACGACGCTCTTCCGATCT**TAGCGTGAAGACGACAGAACCAATGCTGG GATCCATTATCGGCGGCGAATTTACCACCATTGAAAACCAGCCGTGGTTTGCGGCGATTTATCGTCGTCATCGTGGCGGCAGCGTGA CCTATGTGTGCGGCGGCAGCCTGATTAGCCCGTGCTGG**AGATCGGAAGAGCACACGTCTGAACTCCAGTCACATCACGATCTCGTAT GCCGTCTTCTGCTTG**
Subvariant 1	TAGCGTGAAGACGACAGAACCATCAGTGGGATCCATTATCGGCGGCGAATTTACCACCATTGAAAACCAGCCGTGGTTTGCGGCGAT TTATCGTCGTCATCGTGGCGGCAGCGTGACCTATGTGTGCGGCGGCAGCCTGATTAGCCCGTGCTGG
Subvariant 2	TAGCGTGAAGACGACAGAACCACTCATGGGATCCATTATCGGCGGCGAATTTACCACCATTGAAAACCAGCCGTGGTTTGCGGCGAT TTATCGTCGTCATCGTGGCGGCAGCGTGACCTATGTGTGCGGCGGCAGCCTGATTAGCCCGTGCTGG
Subvariant 3	TAGCGTGAAGACGACAGAACCAGCAATGGGATCCATTATCGGCGGCGAATTTACCACCATTGAAAACCAGCCGTGGTTTGCGGCGAT TTATCGTCGTCATCGTGGCGGCAGCGTGACCTATGTGTGCGGCGGCAGCCTGATTAGCCCGTGCTGG
Subvariant 4	TAGCGTGAAGACGACAGAACCATGACTGGGATCCATTATCGGCGGCGAATTTACCACCATTGAAAACCAGCCGTGGTTTGCGGCGAT TTATCGTCGTCATCGTGGCGGCAGCGTGACCTATGTGTGCGGCGGCAGCCTGATTAGCCCGTGCTGG
Subvariant 5	TAGCGTGAAGACGACAGAACCACAAGTGGGATCCATTATCGGCGGCGAATTTACCACCATTGAAAACCAGCCGTGGTTTGCGGCGAT TTATCGTCGTCATCGTGGCGGCAGCGTGACCTATGTGTGCGGCGGCAGCCTGATTAGCCCGTGCTGG
Subvariant 6	TAGCGTGAAGACGACAGAACCATACTTGGGATCCATTATCGGCGGCGAATTTACCACCATTGAAAACCAGCCGTGGTTTGCGGCGAT TTATCGTCGTCATCGTGGCGGCAGCGTGACCTATGTGTGCGGCGGCAGCCTGATTAGCCCGTGCTGG
Subvariant 7	TAGCGTGAAGACGACAGAACCAGAACTGGGATCCATTATCGGCGGCGAATTTACCACCATTGAAAACCAGCCGTGGTTTGCGGCGAT TTATCGTCGTCATCGTGGCGGCAGCGTGACCTATGTGTGCGGCGGCAGCCTGATTAGCCCGTGCTGG
Subvariant 8	TAGCGTGAAGACGACAGAACCAACTGTGGGATCCATTATCGGCGGCGAATTTACCACCATTGAAAACCAGCCGTGGTTTGCGGCGAT TTATCGTCGTCATCGTGGCGGCAGCGTGACCTATGTGTGCGGCGGCAGCCTGATTAGCCCGTGCTGG
Subvariant 9	TAGCGTGAAGACGACAGAACCATATCTGGGATCCATTATCGGCGGCGAATTTACCACCATTGAAAACCAGCCGTGGTTTGCGGCGAT TTATCGTCGTCATCGTGGCGGCAGCGTGACCTATGTGTGCGGCGGCAGCCTGATTAGCCCGTGCTGG

Table S2. Oligonucleotides used for libraries preparation. All oligonucleotides were synthesized on Applied Biosystems ABI 3900 and gel-purified.

**linear amplification**	
TruSeqNNNtestpol	ACACGACGCTCTTCCGATCTNNNNNNNNNNNNNNTAGCGTGAAGACGACAGAACCA
**1st PCR amplification**	
TruSeq PCR Uni-short-21	TACACGACGCTCTTCCGATCT
TruSeq Rev testpol Bridge	GTGACTGGAGTTCAGACGTGTGCTCTTCCGATCTCCAGCACGGGCTAATCAGGCT
**2nd PCR amplification**	
TruSeq Universal long	AATGATACGGCGACCACCGAGATCTACACTCTTTCCCTACACGACGCTCTTCCGATCT
TruSeq Rev long Index	CAAGCAGAAGACGGCATACGAGATACATCGGTGACTGGAGTTCAGACGTGTGCTCTTCCGATCT

Table S3. Amplification parameters. Parameters that vary across replicates are shown with dash.

Sample	Template DNA, ng	1st PCR, cycles	2nd PCR, cycles*	2nd PCR, cycles**	Denaturation T, °C
Encyclo	0.02	20	22	14	95
Tersus-buffer1	0.02	20	24/22	14	95
Tersus-buffer2	0.02	20	22/24	14	95
SNP-detect	0.02	20	25/24	17	95
SD	0.02	20	23/22	15	92
Kapa HF	0.04	20	25/22	14	95
Phusion	0.1	25	29	18	98
TruSeq	0.02	20	25	17	95
Taq-HS	0.02	20	25	15	95
KTN	0.02	20	22/23	14	95

* 1st PCR errors assay with a sampling bottleneck.

** linear amplification errors assay

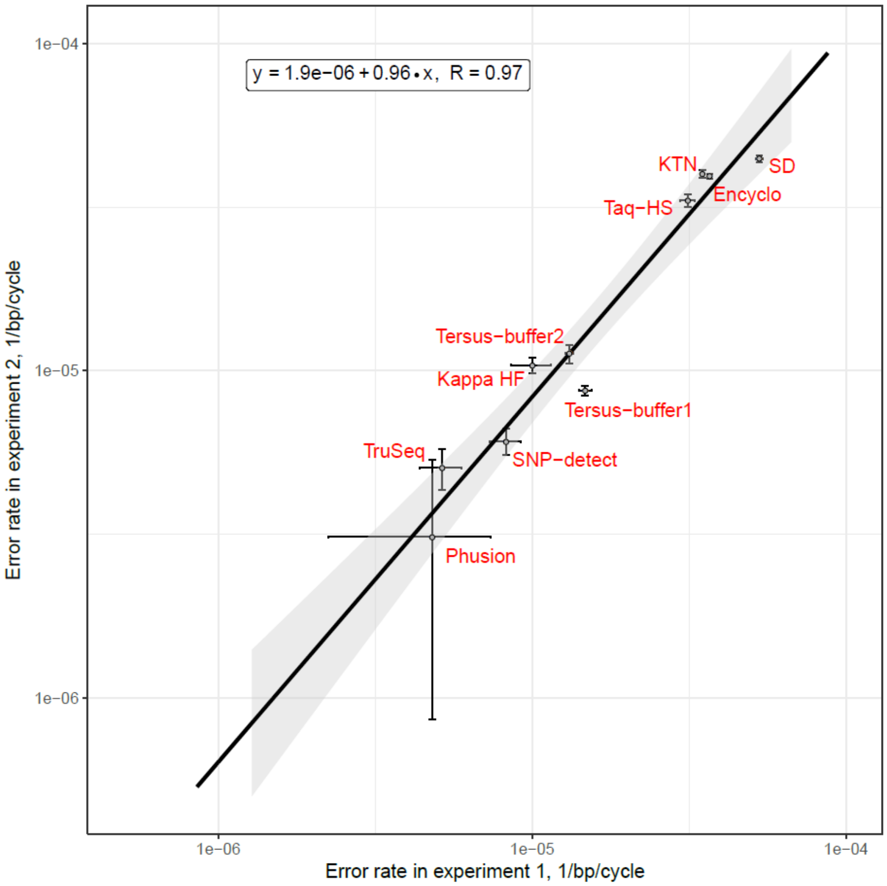

Supplementary Figure 1. Consistency of error rate estimates between two independent experiments. Scatter-plot comparing PCR error rate as identified in first and second experiment for each sample analyzed in present study. PCR error rates were normalized by total number of cycles of 1st PCR and the total number of bases in template. Error bars represent binomial confidence intervals for values obtained in first and second experiment.

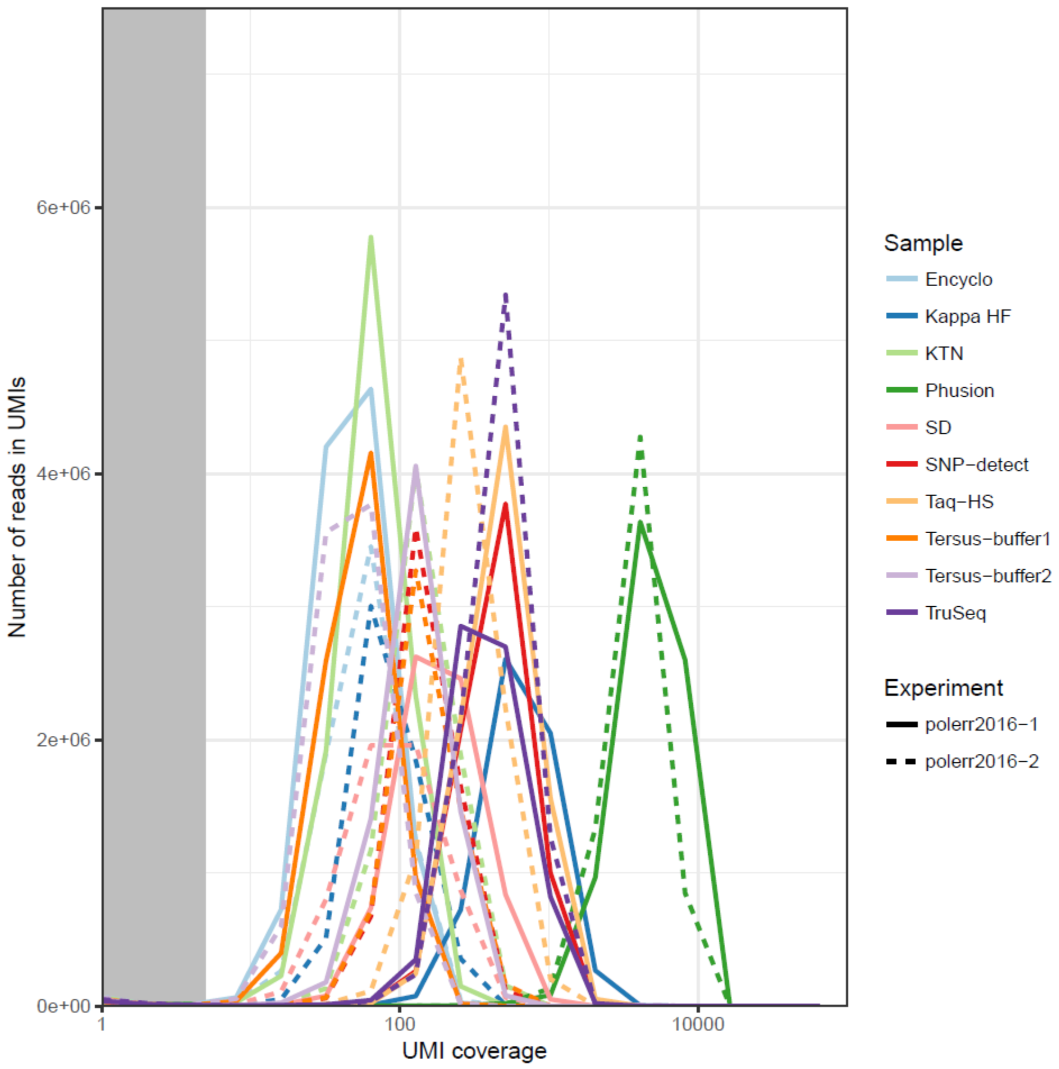

Supplementary Figure 2. UMI coverage distribution for each sample and experiment. Shaded line shows UMIs that were discarded as they do not have enough coverage (<5 reads per UMI) to ensure correction of 2nd PCR and sequencing errors. Note that all samples show a characteristic peak clearly separated from low-coverage zone.

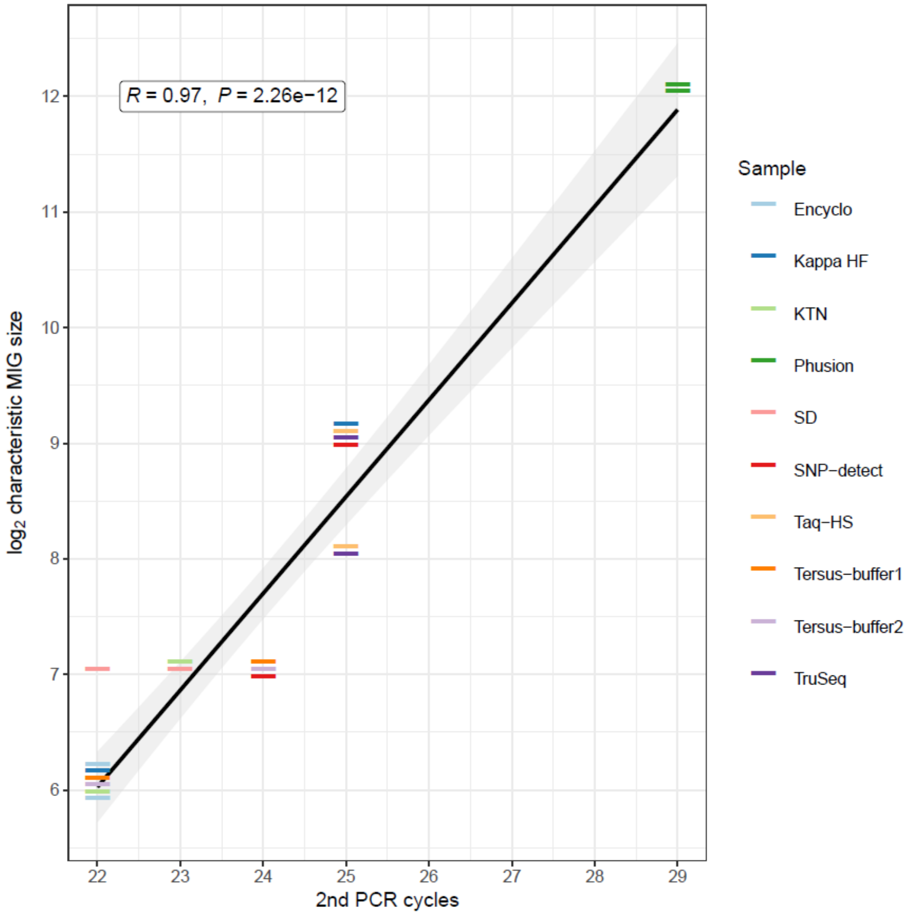

Supplementary Figure 3. Consistency between UMI coverage and number of 2nd stage PCR cycles. The median number of reads per UMI sequence in first and second experiment is plotted against number of cycles used for 2nd PCR for each polymerase. A random jitter was added to allow distinguishing different polymerases (note: correlations and linear fit were computed using raw values).
